# ASAP-IIOT: An Anonymous Secure Authentication Protocol for Industrial Internet of Things

**DOI:** 10.3390/s24041243

**Published:** 2024-02-15

**Authors:** Na Li, Maode Ma, Hui Wang

**Affiliations:** 1 School of Computer Science, Zhejiang Normal University, Jinhua 321000, China; lunana@zjnu.edu.cn; 2 College of Engineering, Qatar University, Doha P.O. Box 2713, Qatar; acadmmd@gmail.com

**Keywords:** industrial internet of things (IIoT), security, authentication, elliptic curve cryptography

## Abstract

With the increasing demand for a digital world, the Industrial Internet of Things (IIoT) is growing rapidly across various industries. In manufacturing, particularly in Industry 4.0, the IIoT assumes a vital role. It encompasses many devices such as sensing devices, application servers, users, and authentication servers within workshop settings. The security of the IIoT is a critical issue due to wireless networks’ open and dynamic nature. Therefore, designing secure protocols among those devices is an essential aspect of IIoT security functionality and poses a significant challenge to the IIoT systems. In this paper, we propose a lightweight anonymous authentication protocol to preserve privacy for IIoT users, enabling secure IIoT communication. The protocol has been validated to demonstrate its comprehensive ability to overcome various vulnerabilities and prevent malicious attacks. Finally, the performance evaluation confirms that the proposed protocol is more effective and efficient than the existing alternatives.

## 1. Introduction

The Industrial Internet of Things (IIoT) is the incorporation of IoT technologies into industrial sectors, linking an extensive array of industrial sensors, devices, and user endpoints to generate significant volumes of industrial data [1,2]. The amount of data produced in the IIoT is directly linked to the growing number of internet-connected devices [3,4,5]. Typical application scenarios encompass smart manufacturing, logistics management, and remote equipment maintenance. These typical application scenarios represent critical components of modern industrial systems, where ensuring secure and efficient communication between devices has become paramount. The primary application scenario for our protocol is in smart manufacturing, specifically for supervisorial management of production lines. An observation system for managing production lines is the subject of investigation, encompassing various devices such as sensors, application servers, and users. Within this system, numerous sensors, integrated into a production line, gather data on operational parameters and status, transmitting this information to the corresponding application server. The manager of production lines is a user in the system monitoring the operation of some production lines by retrieving the operation information stored at the corresponding application servers. Throughout the overarching management procedure, to uphold accountability and privacy, the system must guarantee that only authorized managers can access information pertaining to specified production lines. Unauthorized users are barred from accessing any server information. Furthermore, only servers supervised by a particular authenticated user are accessible for data retrieval. Of course, this system can also be utilized in logistics management and remote equipment maintenance.

Within the IIoT realm, wireless sensor networks (WSNs) play a primary role in connecting extensive sensor nodes. Besides key management, user authentication serves as a fundamental security mechanism to confirm the user’s validity in WSNs [6,7]. For applications requiring swift data collection, like remote pipeline monitoring, application servers gather data from sensing devices, enabling users to access these data through mobile devices. A recent study conducted by Sobin [8] underscores that scalability, the standardization of architectures and protocols, energy efficiency, and security and privacy remain unresolved challenges that impede the widespread implementation of an IIoT system. The study in [9,10] emphasizes that IoT systems face numerous privacy and security issues due to their resource-constrained devices, heterogeneous deployments, and dynamic nature. Secure and dependable authentication protocols are demanded for the establishment of mutual trust between the users and servers [11].

Ensuring the confidentiality, availability, and integrity of critical data while securing and maintaining reliable IIoT operations remains a top priority. Several protocols have emerged to complete secure IIoT authentication. A secure ECC-based authentication protocol for IoT edge devices (ECCbAP) has been introduced in [12] to secure the connectivity between edge devices and cloud servers. The results show that the ECCbAP scheme provides a high level of security and is also more resource-efficient. However, it has not proved its ability against replay attacks. Adversaries could potentially compromise systems by repeatedly forwarding the stolen messages, leading to resource exhaustion. In [13], a lightweight wireless sensor network (WSN) authentication protocol with user anonymity has been proposed, offering numerous security features with high efficiency. However, some design vulnerabilities under the desynchronization and smart card stolen attacks still exist, which could result in privacy violations. A provably secure and efficient identity-based anonymous authentication scheme for mobile edge computing (AASM) has been developed in [14], which may introduce excessive latency in IIoT settings despite its demonstrable security functionality. The protocol achieves mutual authentication in only a single message exchange round, as well as ensuring both user anonymity and untraceability. An ECC-based protocol for WBAN systems (ECCPWS) has been introduced in [15] to secure wireless body area network (WBAN) communications, but its registration is too idealistic without the feasibility for implementation and an absolutely secure channel for registration. In [16,17], two bilinear pairing-based protocols have been presented to address the privacy concerns in IIoT, but they come with significant computational complexity. A lightweight AES encryption algorithm to safeguard sensing data transmitted through the Internet from potential attacks has been proposed in [18,19]; however, these protocols are vulnerable to impersonation attacks. A secure authentication protocol for IoT and cloud servers (SAPIC) based on elliptic curve cryptography (ECC) has been designed in [20], which has demonstrated its robustness by the extended Canetti–Krawczyk adversary model. However, the protocol exhibits the absence of perfect secrecy and faces challenges related to clock desynchronization. Furthermore, a massive IoT device attestation protocol has been proposed in [21] leveraging reputation and physically unclonable functions, which is not suitable to work in IIoT with user terminals. A secure mutual authentication protocol for the IoT environment (ASMAP) has been introduced in [22]; this protocol is capable of resisting impersonation attacks, replay attacks, and password-guessing attacks. However, it is susceptible to modification attacks.

The advantages and disadvantages of the most relevant authentication schemes for the proposed protocol are summarized in Table 1.

In summary, the above-mentioned solutions exhibit vulnerabilities to replay attacks and traceability attacks without privacy protections. Additionally, they suffer from superfluous parameters and other deficiencies. The existing protocols struggle to achieve a balance among authentication efficiency, security, and anonymity. To overcome those shortcomings, in this paper, we design a novel secure authentication protocol to enhance security functionality and authentication efficiency in an IIoT scenario, where a production line manager monitors the operation of a few production lines by accessing the data collected and stored at the corresponding application servers. The primary contributions produced in this paper can be summarized as follows.

- An anonymous secure authentication protocol for IIoT (ASAP-IIOT) to implement mutual authentication between the application servers and users.
- The ASAP-IIOT provides the capability to ensure user anonymity in secure communication. It utilizes both asymmetric and symmetric encryption to ensure the secure reception of user information by servers.
- The ASAP-IIOT has demonstrated its logic correctness by using BAN Logic and been formally verified for its security functionality by using Scyther Tool. Moreover, it outperforms some existing solutions in terms of computational costs, communication costs, and total authentication overhead evaluated by simulation experiments.

The remainder of this paper is structured as follows. In Section 2, the system model under study is introduced. In Section 3, the preliminaries relevant to this work are introduced, and Section 4 delineates the details of the proposed ASAP-IIOT. In Section 5 and Section 6, the security evaluation and performance evaluation are presented, respectively, with their results to show the advantages of the ASAP-IIOT. Finally, Section 7 concludes the paper with a summary.

## 2. System Model

### 2.1. System Model of the Proposal

The system under study is an observation system for production line management with multiple types of devices including sensors, application servers, and users. In the system, multiple sensors equipped with a production line collect information on the parameters and status of the operation and send the information to the associated application server, which controls the operation of the production line and keeps the complete information of the production line. There could be multiple such production lines existing in the system. The manager of production lines is a user in the system monitoring the operation of some production lines by retrieving the operation information stored at the corresponding application servers. The manager needs to react to some faults that occur in the system. In the overall management process, to preserve responsibility and privacy, the system should ensure that only the valid manager can access the information on the operation of some specified production lines. Invalid users should not be able to obtain any information on any server. Moreover, only the servers supervised by a particular authenticated user should be accessed by him for the retrieval of data. To achieve this goal, mutual authentication is highly demanded.

**Figure 1:**
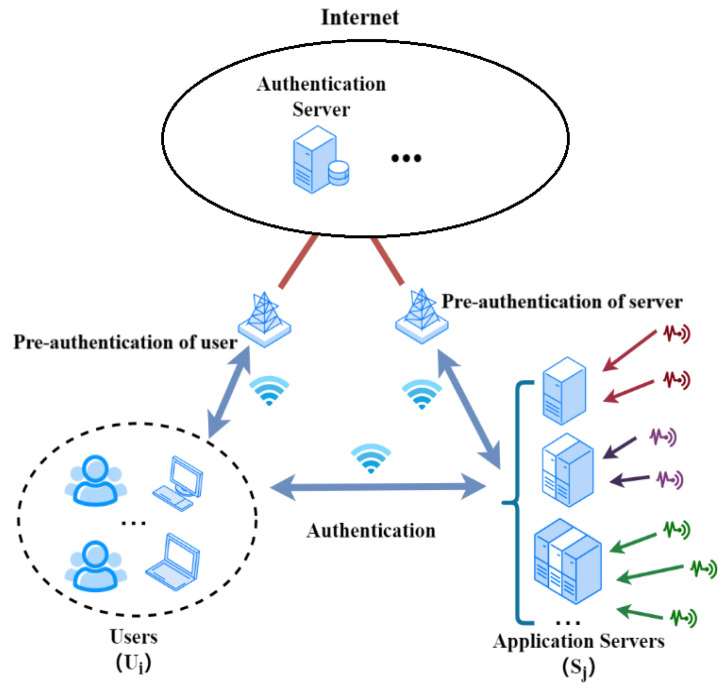
System Model.

With this consideration, we design our detailed system model as shown in Figure 1. In the model, the major components include the application server *S_j_*, the user *U_i_*, and the authentication server *AS*.

Application Server, *S_j_*: Each *S_j_* controls all of the sensors in one production line. The information collected by the sensors will be stored and primarily processed by the server for further analysis and retrieval. The information on the production line may include system parameters, production volumes, the overall length of the production period, the amount of products, etc. *S_j_* should be registered or pre-authenticated to the system by the *AS* before offering services to ensure their validity.

User, *U_i_*: The *U_i_* is a supervisor of one or a few production lines who is able to access the real-time information of the production lines collected and stored by the sensors at particular servers. A specific *U_i_* can access one or more specific *S_j_*. The *U_i_* should be registered or pre-authenticated on the system by the *AS* before starting the function to ensure their validity.

Authentication Server, *AS*: The *AS* works for the system authentication purposes responsible for the pre-authentication of users and application servers when they are connected to the system before they function. The *AS* will generate and distribute necessary confidential information such as keying materials for each user and each application server.

The communication between the *AS* and users as well as between the *AS* and application servers for the pre-authentication is achieved by the long-term evolution (LTE) of 4G wireless communication technology and the Internet, while the communication between users and servers for the mutual authentication and information retrievals is completed by the device-to-device communication in the 4G wireless networks.

### 2.2. Adversary Model

In this study, the widely adopted Dolev-Yao [23] adversary model is leveraged to present the capacity of the adversary in the system, whereby communication between entities over an insecure channel with untrustworthy end devices is presumed. Under this model, adversary *A* is posited to have the capabilities to fully control the communication channel. Succinctly, *A* can intercept, alter, retransmit, fabricate, and erase any data conveyed over the insecure network.

## 3. Preliminaries

This section provides a brief overview of fundamental concepts and technical preliminaries utilized in crafting the proposed protocol, including ECC and the Burrows–Abadi–Needham (BAN) logic.

### 3.1. Elliptic Curve Cryptography

The ECC [24,25] works based on algebraic concepts related to elliptic curves. Let q > 3 be a prime over the prime finite fields $Z_{q}=\{0,\;1,\;\ldots ,\;q-1\}$; the elliptic curve ${y\;}^{2}{=x\;}^{3}+ax+b$ is the set of $E_{q}\;(a,\;b)$ of solutions $(x,\;y)\;\in {\;Z}_{q}{\times \;Z}_{q}$ to the congruence $y^{2}{=x}^{3}+ax+b\;(\mathrm{mod}\;q)$, where $a,\;b\;\in {\;Z}_{q}$ are constants with the non-singularity of the elliptic curve property ${4a}^{3}{+27b}^{2}\;\neq \;0\;(\mathrm{mod}\;q)$. This equation is the Weierstrass equation [26,27,28].

The security of ECC relies on the intractability of the elliptic curve discrete logarithm problem (ECDLP). Let P, Q∈(a, b) be two points such that Q=k · P, where *k* is a positive integer. In fact, the smallest positive integer *k* with this property is called the discrete logarithm *Q* at base *P* [28,29]. For the ECDLP, given *k* and *P*, computing *Q* is computationally facile. But determining *k* given *Q* and *P* is infeasible for sufficiently large *q*. In other words, an adversary would expend substantial computational time and resources to leverage points *P* and *Q* to derive *k*.

Building upon elliptic curves and the ECDLP, the security of ECC can be characterized by the elliptic curve computational Diffie–Hellman problem (ECCDHP). Let GE be the cyclic group generated by the base point *G* and the operation rules of Abelian groups on the elliptic curve *E*(*a*, *b*). Generally, elliptic curve points denote public keys, while positive integers signify private keys. To conserve memory and bandwidth, point compression techniques should be contemplated.

### 3.2. BAN Logic

BAN logic has emerged as a routine and efficacious methodology for analyzing authentication protocols. Underpinned by logical rules, this approach can ascertain the trustworthiness of exchanged information against malicious nodes. Generally, the inference process in BAN logic encompasses four key steps: (1) idealize the protocol model, (2) develop the initial assumptions, (3) set the specific test goals, and (4) employ the initial hypotheses and logical rules to execute the formal analysis. The notations used in the BAN logic are as follows:

*P|≡ X:* Principal *P* believes a statement *X*, or *P* is entitled to believe *X*.

#(*X*): Formula *X* is fresh.

*P|⇒ X: P* has jurisdiction over a statement *X*.

*P* ⊲ *X*: *P* sees *X*, the principal *P* receives the message containing *X*, and *P* can read or repeat *X*.

*P|∼ X: P* once said *X*; the principal *P* sent a message containing *X* at some time.

(*X*, *Y*): The formula *X* or *Y* is one part of the formula (*X*, *Y*).

${\left\{X\right\}}_{K}$: The formula *X* is encrypted using the key *K*.

*P*$\overset{K}{↔}$*Q: P* and *Q* may use the shared key *K* to communicate. The key *K* is good in that it will never be discovered by any principal except *P* and *Q*.

${P\;}_{\;⇌}^{\;K}\;$*Q: K* is a shared secret key between *P* and *Q*.

#### Rules of BAN Logic

The BAN logic holds 19 logical rules. Here, we only list six rules used in this paper.

Rule 1: Message Meaning Rule

$$\frac{\;P\;|\equiv {\;P\;}_{⇌\;}^{K}\;Q,\;P\;⊲{\;\{X\}}_{K}}{P\;\left|\equiv \;Q\;\right|\sim \;X}$$

This rule states that if *P* believes that the key *K* is the public key of *Q* and sees *X* encrypted under *Q*’s private key, then *P* believes that *Q* once said *X*.

Rule 2: Nonce Verification Rule

$$\frac{P\;|\equiv \;\#(X),\;P\;|\equiv \;Q\;|\sim \;X}{P\;\left|\equiv \;Q\;\right|\equiv \;X}$$

This rule states that if *P* believes that *X* is fresh and that *Q* once said *X*, then *P* believes that *Q* believes *X*.

Rule 3: Jurisdiction Rule

$$\frac{P\;\left|\equiv \;Q\;\Rightarrow \;X,\;P\;\right|\equiv \;Q\;|\equiv \;X}{P\;|\equiv \;X}$$

This rule states that if *P* believes that *Q* has jurisdiction over X and *P* trusts *Q* on the truth of *X*, then *P* believes *X*.

Rule 4: Freshness Rule

$$\frac{P\;|\equiv \;\#\left(X\right)}{P\;|\equiv \;\#\left(X,\;Y\right)}$$

This rules states that if *P* believes that one part *X* of a formula is fresh, then the entire formula (*X*, *Y*) must also be fresh.

Rule 5: Belief Rule

$$\frac{P\;\left|\equiv \;X,\;P\right|\equiv \;Y}{P\;|\equiv \;\left(X,\;Y\right)}$$

This rules states that if the agent *P* believes the messages *X* and *Y* distinctly, then *P* believes the combined formula (*X*, *Y*).

Rule 6: Send Rules

$$\frac{P\;\left|\equiv Q\right|∼\left(X,Y\right)}{P\;\left|\equiv Q\right|\sim X}$$

This rule states that if *P* believes the agent *Q* said (*X*, *Y*), then it is deduced that *P* also believes the agent *Q* once said *X*.

## 4. The ASAP-IIOT

The survey on the existing literature reveals that certain referenced authentication protocols exhibit vulnerabilities to passive insider secret disclosure and replay attacks. Additionally, these protocols overlook privacy protections and remain susceptible to traceability incursions. Through further investigation, superfluous parameters and other deficiencies across different phases of an authentication process can be identified. Therefore, in this research, a new lightweight authentication protocol is designed to overcome the above-mentioned shortcomings. It cannot only provide security protection but also hold authentication efficiency.

The proposed ASAP-IIOT works in four phases, including initialization, pre-authentication of servers, pre-authentication of users, and authentication. During the initiation of the system, an initialization phase is necessary to publish protocol parameters. When a new production line has been installed, pre-authentication of the server is required to prevent malicious servers from stealing user information. Similarly, when a new manager joins, pre-authentication of the user is required to ensure a valid user. When a user accesses server information, mutual authentication is required to ensure a valid user only accesses the servers under his supervision, resulting in the generation of temporary session keys. A summary of the notations used in this paper is provided in Table 2.

### 4.1. Initialization Phase

Before the system works, the *AS* needs to perform the necessary parameter initialization operations. In this phase, the *AS* publishes protocol parameters, which are listed in Table 2, by performing the following steps.

1. The *AS* selects a hash function such as *h*:{0, 1} × G_E_ → Z_q_*.
2. The *AS* selects its private/public keys as (*SK_AS_*, *PK_AS_*), where *PK_AS_* = *SK_AS_*⋯*G* and *SK_AS_* ∈ Z_q_*.
3. Finally, the *AS* generates and publishes the protocol parameters, i.e., {*G_E_*, *G*, *h*, *PK_AS_*}.
4. The user *U_i_* selects its private/public keys as (*SK_U_*, *PK_U_*), where *PK_U_* = *SK_U_*⋯*G*.
5. The *S_j_* selects its private/public keys as (*SK_S_*, *PK_S_*), where *PK_S_* = *SK_S_*⋯*G*.

### 4.2. Pre-Authentication of Server Phase

**Figure 2:**
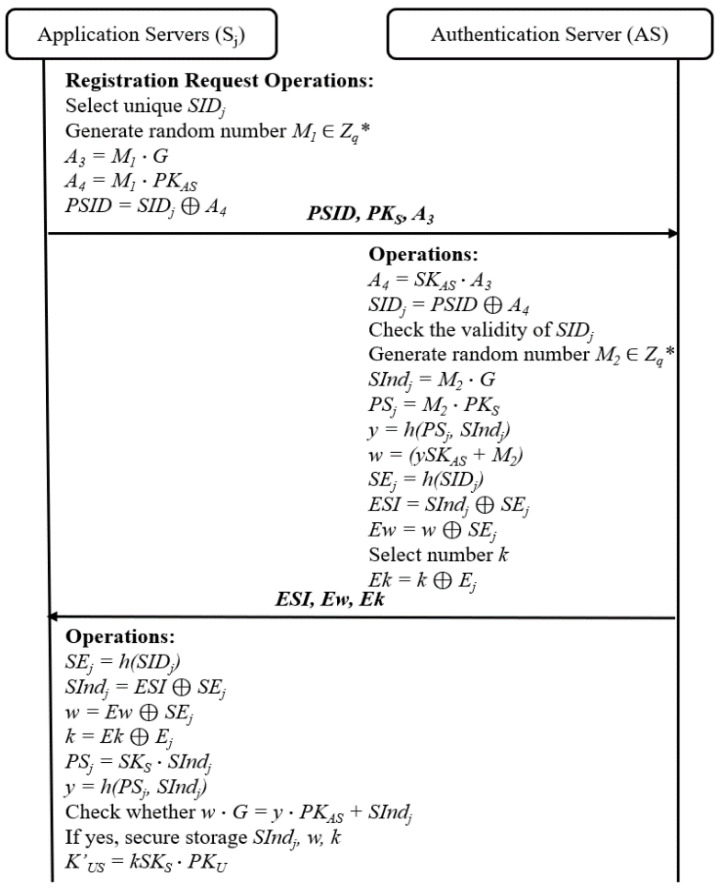
Pre-authentication of server by the ASAP-IIOT.

According to the ASAP-IIOT scheme, each server of a production line must be registered on the *AS* before service. This step ensures the legitimacy of the *S_j_*. The pre-identity verification of servers guarantees the validity of the servers. The pre-identity verification of the servers is completed through the following steps, as illustrated in Figure 2:

1. The server *S_j_* chooses its *SID_j_* and public key *PK_S_*. Then, the *S_j_* generates a random number *M_1_* to randomize its *SID_j_* and calculates *PSID*, *A*_3_*,* and *A*_4_*,* where *A*_3_ = *M*_1_⋯*G*, *A*_4_ = *M*_1_⋯*PK_AS_*_,_ and ${\mathrm{PSID}=\mathrm{SID}}_{j}⊕\;A_{4}$. Then, the *S_j_* sends the request {*PSID*, *PK_S_*, *A*_3_} to the *AS* via an open channel.
2. Once the request message is received, the *AS* calculates *A_4_* and *SID_j_* according to *A*_4_ = *SK_AS_*⋯*A*_3_, and *SID_j_* = *PSID* ⊕ *A*_4_. Then, the *AS* verifies the validity of the message *SID_j_* and picks out a random number *M*_2_∈Z_q_*. Next, it computes *SInd_j_* = *M*_2_⋯*G*, *PS_j_* = *M*_2_⋯*PK_S_*, *y* = *h*(*PS_j_*, *SInd_j_*), and *w* = *ySK_AS_* + *M*_2_, and selects number *k*, which is a value generated by the *AS* known only to the pre-authenticated application servers and users during the authentication process. The *AS* calculates *SE_j_* = *h*(*SID_j_*) and encrypts *SInd_j_*, *w*, and *k* with *SE_j_* by *ESI* = *SInd_j_* ⊕ *SE_j_*, *Ew* = *w* ⊕ *SE_j_*, and *Ek* = *k* ⊕ *SE_j_*. Finally, the *AS* sends {*ESI*, *Ew*, *Ek*} to the server *S_j_* over a public channel.
3. After the message {*ESI*, *Ew*, *Ek*} is received, the *S_j_* calculates *SE_j_* = *h*(*SID_j_*) first. Then, it computes *SInd_j_* and *w* as *SInd_j_* = *ESI* ⊕ *SE_j_*, *w* = *Ew* ⊕ *SE_j_*, and *k* = *Ek* ⊕ *SE_j_*. The server *S_j_* further computes *PS_j_* = *SK_S_* ∙ *SInd_j_* and *y* = *h*(*PS_j_*, *SInd_j_*). After that, the server *S_j_* checks whether *w*⋯*G* = *y*⋯*PK_AS_* + *SInd_j_*. If they are equal, the server *S_j_* stores the message {*SInd_j_*, *w*, *k*} in its database safely and then computes *K_US_* = *kSK_S_*⋯*PK_U_*, where *K_US_* is the shared secret key between the user *U_i_* and the server *S_j_*.

### 4.3. Pre-Authentication of User Phase

**Figure 3:**
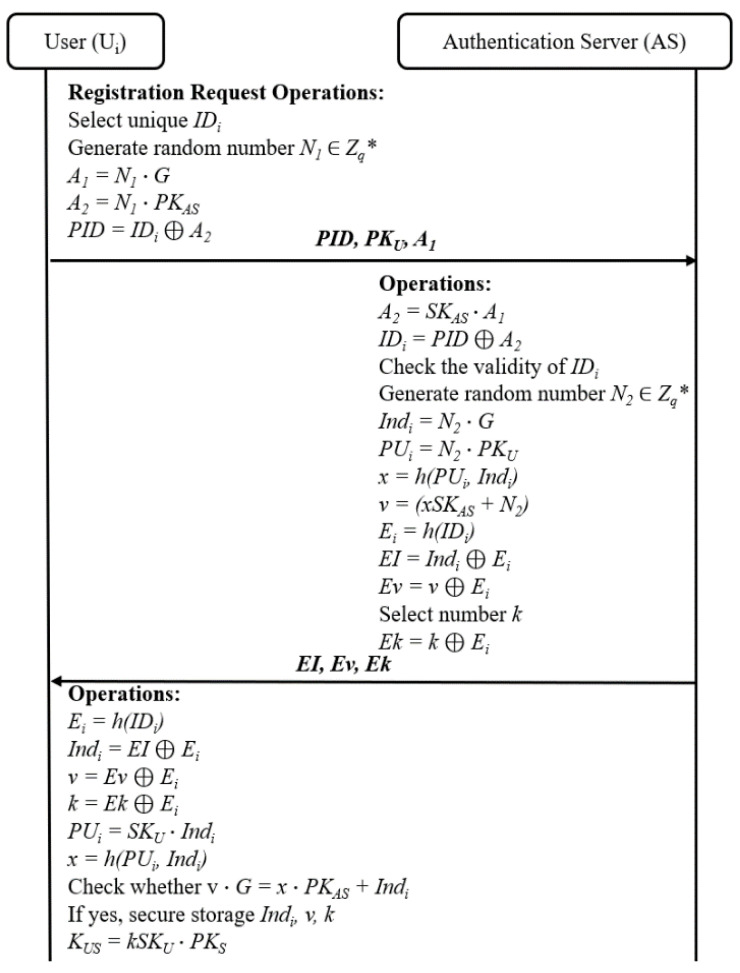
Pre-authentication of user by the ASAP-IIOT.

According to the ASAP-IIOT scheme, every user *U_i_* must be registered on the *AS* before accessing information from the application server *S_j_*. This step ensures the legitimacy of *U_i_* and enables the pre-authentication of the user to be conducted through a public channel. The pre-authentication of the user phase is completed through the following steps as depicted in Figure 3:

1. The user *U_i_* chooses its *ID_i_* and public key *PK_U_*. Then, the *U_i_* generates a random number *N*_1_ to randomize its *ID_i_* and calculates *PID*, *A*_1_, and *A*_2_, where *A*_1_ = *N*_1_⋯*G*, *A*_2_ = *N*_1_⋯*PK_AS_*_,_ and $\mathrm{PID}=\mathrm{IDi}\;⊕\;A_{2}$. Then, the *U_i_* sends the request {*PID*, *PK_U_*, *A*_1_} to the *AS* via an open channel.
2. Once the request message is received, the *AS* calculates *A*_2_ and *ID_i_* according to *A*_2_ = *SK_AS_*⋯*A*_1_, and *ID_i_* = *PID* ⊕ *A*_2_. Then, the *AS* verifies the validity of the message *ID_i_* and picks out a random number *N*_2_∈Z_q_*. Next, it computes *Ind_i_* = *N*_2_⋯*G*, *PU_i_* = *N*_2_⋯*PK_U_*, *x* = *h*(*PU_i_*, *Ind_i_*), and *v* = *xSK_AS_* + *N*_2_, and selects the number *k*, which is a value generated by the *AS* known only to the pre-authenticated application servers and users during the authentication process. The *AS* calculates *E_i_* and encrypts *Ind_i_*, *v*, and *k* with *E_i_* by *EI* = *Ind_i_* ⊕ *E_i_*, *Ev* = *v* ⊕ *E_i_*, and *Ek* = *k* ⊕ *E_i_*. Finally, the *AS* sends {*EI*, *Ev*, *Ek*} to the user *U_i_* over a public channel.
3. After the message {*EI*, *Ev*, *Ek*} is received, the *U_i_* calculates *E_i_* = *h*(*ID_i_*) first. Then, it computes *Ind_i_* and *v* as *Ind_i_* = *EI* ⊕ *E_i_*, *v* = *Ev* ⊕ *E_i_*, and *k* = *Ek* ⊕ *E_i_*. The user *U_i_* further computes *PU_i_* = *SK_U_*⋯*Ind_i_* and *x* = *h*(*PU_i_*, *Ind_i_*). After that, the user *U_i_* checks whether *v*⋯*G* = *x*⋯*PK_AS_* + *Ind_i_*. If they are equal, the user *U_i_* stores the message {*Ind_i_*, *v*, *k*} in its database safely and then computes *K_US_* = *kSK_U_* ∙ *PK_S_*, where *K_US_* is the shared secret key between the user *U_i_* and the *S_j_*. It is obvious that the *S_j_* computes this session key as *K’_US_* = *kSK_S_*⋯*PK_U_*, which is equal to the computed key on the user side.

### 4.4. Authentication Phase

**Figure 4:**
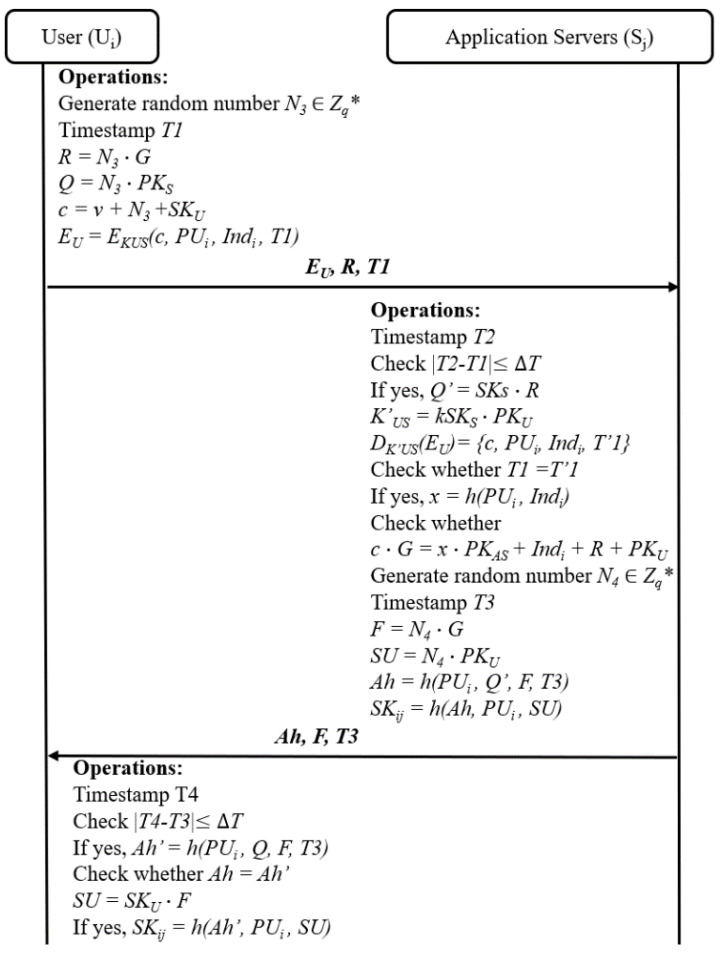
Authentication phase of the ASAP-IIOT.

When the user *U_i_* wants to access data from the *S_j_*, the user and server need to complete mutual identity authentication and key negotiation. The authentication phase is completed in the following steps as depicted in Figure 4. If a manager is in charge of *m* production lines, he needs to perform this mutual authentication for *m* rounds.

1. The user *U_i_* selects a random number *N*_3_ ∈ Z_q_*, calculates *Q*, *R*, and *c*, and then computes *E_U_* = *E_Kus_* (*c*, *PU_i_, Ind_i_*, *T*1). Next, it sends the message of authentication request {*E_U_*, *R*, *T*1} to the server *S_j_*.
2. After the message {*E_U_*, *R*, *T*1} is received by the server *S_j_* at time *T*2, the server *S_j_* checks the timestamp *T*1 using the inequality |*T*2 − *T*1| ≤ ∆*T*. If it is not established, the *S_j_* will reject the request. Otherwise, the *S_j_* approves *T1* and goes to the next step to compute *Q*′, *K*′*_US_*, and {*c*, *PU_i_*, *Ind_i_*, *T*′1}. Then, the server *S_j_* checks whether *T*1 = *T*′1. If it is not equal, the session will end. Otherwise, the *S_j_* recognizes the user *U_i_* as a valid user to compute *x* = *h*(*PU_i_*, *Ind_i_*) and verifies whether *c∙G* = *xPK_AS_* + *R* + *PK_U_* + *Ind_i_*. If the equation does not hold, the session will end. Otherwise, the *S_j_* selects a random number *N*_4_ ∈ Z_q_* and calculates *F* = *N*_4_ ∙ *G*, *SU* = *N*_4_ ∙ *PK_U_, Ah* = *h*(*PU_i_*, *Q*, *F*, *T*3), and *SK_ij_* = *h*(*Ah*, *PU_i_*, *SU*) as the session key. Finally, the server *S_j_* sends the {*Ah*, *F*, *T*3} to the *U_i_* in response to the authentication request.
3. After the message {*Ah*, *F*, *T*3} is received by the *U_i_* at time *T*4, the user *U_i_* checks the inequality |*T*4 − *T*3| ≤ ∆*T*. If it does not hold, the user *U_i_* will reject the response of the server *S_j_*. Otherwise, the *U_i_* computes *Ah*′ = *h*(*PU_i_*, *Q*, *F*, *T*3). Then, it verifies whether *Ah*′ = *Ah*. If yes, the *U_i_* finds the server *S_j_* as a valid server and computes *SU* = *SK_U_*⋯*F* and *SK_ij_ = h*(*Ah*′, *PU_i_*, *SU*) as the session key. Since *SK_U_*⋯*F* = *N*_4_⋯*PK_U_* = *SU,* it is obvious the generated session key on the *U_i_* side is equal to the generated session key on the *S_j_* side.

## 5. Security Evaluation

In this section, we commence with a logical correctness proof to assess the ASAP-IIOT scheme by using BAN logic. Subsequently, the ASAP-IIOT is formally verified by using the Scyther tool. Finally, a qualitative security analysis of the proposed protocol is performed to demonstrate its success in meeting the security objectives.

### 5.1. The Logic Correctness Proof by BAN Logic

In this subsection, we use BAN logic to prove the logic correctness of the ASAP-IIOT scheme. Due to the similarity between the pre-authentication of user and the pre-authentication of server, and considering that user entry and exit from the system are more frequent, we will only prove the pre-authentication of user process and the mutual authentication process in this context. The two authentication processes are idealized in BAN logic first. Then, reasonable assumptions are made with expected security goal setting. And finally, we reason and infer whether these security goals can be achieved.

1. Establishment of the idealized protocol model

In order to idealize the ASAP-IIOT scheme into a BAN logic model, irrelevant components should be ignored. In the model, *U_i_*, *S_j_*, and *AS* are considered the principal U_i_ (1 ≤ *i* ≤ *n*), *S_j_* (1 ≤ *j* ≤ *m*), and *AS*, respectively. The generic forms of ASAP-IIOT are provided below:

Message 1: $U_{i}\to {\;\mathrm{AS}\;:\;\{\mathrm{PID},\;\mathrm{PK}}_{U}{,\;A}_{1}\}.$

Message 2: ${\mathrm{AS}\to U_{i}\;:\;\{\mathrm{Ind}}_{i}{,\;v,\;k\;\}}_{E_{i}}$.

Message 3: *U_i_* → *S_j_* : ${\{E}_{U},\;R,\;T1\}$.

Message 4: *S_j_*→ *U_i_*: {Ah, F, T3}.

2. Establishment of the initial assumption

By analyzing the ASAP-IIOT, some initiative assumptions can be obtained. All of these assumptions are based on factual knowledge and are able to facilitate key agreement verification.

**Hypothesis** **1:** *$U_{i}\;|\equiv {\;U}_{i}\;\overset{{\;E}_{i}}{⇌}\;\mathrm{AS}$*.

**Hypothesis** **2:** *$U_{i}\;|\equiv {\;\#(\mathrm{Ind}}_{i},v,\;k)$*.

**Hypothesis** **3:** *$U_{i}\;|\equiv \;\mathrm{AS}\;|\Rightarrow {\;(\mathrm{Ind}}_{i},v,\;k)$*.

**Hypothesis** **4:** *$S_{j}\;|\equiv {\;U}_{i}\;\overset{\;{\mathrm{SK}}_{U}}{⇌}\;S_{j}$*.

**Hypothesis** **5:** *$U_{i}\;|\equiv {\;N}_{3}$*.

**Hypothesis** **6:** *${\;S}_{j}\;|\equiv \;\#(R)$*.

**Hypothesis** **7:** *$S_{j}\;|\equiv {\;U}_{i}|\;\Rightarrow \;R$*.

**Hypothesis** **8:** *$S_{j}\;|\equiv {\;\mathrm{SK}}_{S}$*.

**Hypothesis** **9:** *$S_{j}\;|\equiv {\;\#(\mathrm{PU}}_{i})$*.

**Hypothesis** **10:** *$S_{j}\;|\equiv {\;U}_{i}|\Rightarrow {\;\mathrm{PU}}_{i}$*.

**Hypothesis** **11:** *$S_{j}\;|\equiv \;F$*.

**Hypothesis** **12:** *$S_{j}\;|\equiv \;\#(T4$)*.

**Hypothesis** **13:** *$S_{j}\;|\equiv {\;N}_{4}$*.

**Hypothesis** **14:** *$U_{i}\;|\equiv {\;S}_{j}|∼{\;(\mathrm{PU}}_{i},\;Q\prime ,\;F,\;T3)$*.

**Hypothesis** **15:** *$U_{i}\;|\equiv {\;\#(\mathrm{PU}}_{i},\;Q\prime ,\;F,\;T3)$*.

**Hypothesis** **16:** *$U_{i}\;|\equiv {\;S}_{j}|\Rightarrow \;F$*.

**Hypothesis** **17:** *$U_{i}\;|\equiv \;Q$*.

**Hypothesis** **18:** *$U_{i}\;|\equiv \;R$*.

**Hypothesis** **19:** *$U_{i}|\equiv {\;\mathrm{PU}}_{i}$*.

3. Establishment of the intended purpose

According to the analytic procedures of the BAN logic, the protocol is secure against various malicious attacks only if the intended purpose can be met. In order to complete the authentication proof, the ASAP-IIOT scheme must satisfy the following test goals.

Goal 1: $U_{i}\;|\equiv {\;(\mathrm{Ind}}_{i},\;v,\;k)$.

Goal 2: $S_{j}\;|\equiv {\;\mathrm{SK}}_{\mathrm{ij}}$.

Goal 3: $U_{i}\;|\equiv {\;\mathrm{SK}}_{\mathrm{ij}}$.

4. Protocol analysis

Based on the above-mentioned hypotheses and the logical rules of the BAN logic, we provide the main procedures of proof as follows.

According to Message 2, we obtain the following:

(1) $$U_{i}\;⊲\;\;{\left\{{\mathrm{Ind}}_{i},\;v,\;k\;\right\}}_{E_{i}}\;$$

According to Hypothesis 1 and Equation (1), we employ Rule 1 to derive the following:

(2) $$U_{i}\;|\equiv \;\mathrm{AS}\;|∼{\;(\mathrm{Ind}}_{i},\;v,\;k)$$

According to Equation (2) and Hypothesis 2, we apply Rule 2 to derive the following:

(3) $$U_{i}\;|\equiv \mathrm{AS}\;|\equiv {\;(\mathrm{Ind}}_{i},\;v,\;k)$$

According to Hypothesis 3 and Equation (3), we employ Rule 3 to deduce the following:

(4) $$U_{i}\;|\equiv {\;(\mathrm{Ind}}_{i},\;v,\;k)$$

which satisfies Goal 1.

According to Message 3, we obtain the following:

(5) $$S_{j}\;⊲\;\left\{E_{U},\;R,\;T1\right\}:\{{\left\{{c,\;\mathrm{PU}}_{i}{,\;\mathrm{Ind}}_{i},\;T’1\right\}}_{K_{\mathrm{US}}}{,\;N}_{3}\cdot G,\;T1\}$$

According to Hypothesis 4 and Equation (5), we apply Rule 1 to deduce the following:

(6) $$S_{j}\;|\equiv {\;U}_{i}|∼{\{c,\;\mathrm{PU}}_{i}{,\;\mathrm{Ind}}_{i},\;T’1\}$$

According to Equation (6), we apply Rule 6 to deduce the following:

(7) $$S_{j}\;|\equiv {\;U}_{i}|∼{\;\mathrm{PU}}_{i}$$

According to Equation (5), we apply Rule 6 to deduce the following:

(8) $$S_{j}\;|\equiv {\;U}_{i}|∼{\;N}_{3}$$

According to Equation (5), we apply Rule 6 to deduce the following:

(9) $$S_{j}\;|\equiv {\;U}_{i}|∼\;R$$

According to Hypothesis 6 and Equation (9), we apply Rule 2 to deduce the following:

(10) $$S_{j}\;|\equiv {\;U}_{i}\;|\equiv \;R$$

According to Hypothesis 7, Equations (8) and (10), we apply Rule 2 and Rule 3 to deduce the following:

(11) $$S_{j}\;|\equiv \;R$$

According to Hypothesis 8 and Equation (11), we deduce the following:

(12) $$S_{j}\;|\equiv {\;\mathrm{SK}}_{S}\cdot \;R=Q\prime$$

According to Hypothesis 9 and Equation (7), we apply Rule 2 to deduce the following:

(13) $$S_{j}\;|\equiv {\;U}_{i}|\;\equiv {\;\mathrm{PU}}_{i}$$

According to Hypothesis 10 and Equation (13), we apply Rule 3 to deduce the following:

(14) $$S_{j}\;|\equiv {\;\mathrm{PU}}_{i}$$

According to Hypothesis 11, 12, Equations (10)–(12) and (14), we apply Rule 5 to deduce the following:

(15) $$S_{j}\;|\equiv {\;(\mathrm{PU}}_{i},\;Q’,\;F,\;T3)$$

According to Hypothesis 13 and Equation (10), we deduce the following:

(16) $$S_{j}\;|\equiv {\;N}_{4}\cdot {\mathrm{PK}}_{U}=\mathrm{SU}$$

According to Equation (7) and Equation (16), we deduce the following:

(17) $$S_{j}\;|\equiv \;{\left({{(\mathrm{PU}}_{i},Q\prime ,F,T3)}_{h}{,\;\mathrm{PU}}_{i},\;\mathrm{SU}\right)}_{h}{=\mathrm{SK}}_{\mathrm{ij}}$$

which satisfies Goal 2.

According to Message 3, we obtain the following:

(18) $$U_{i}⊲{\;\{\mathrm{PU}}_{i}\;,\;Q\prime ,\;F,\;T3\}$$

According to Hypothesis 14, 15, and Equation (18), we apply Rule 2 to deduce the following:

(19) $$U_{i}\;|\equiv {\;S}_{j\;}|\equiv {\;(\mathrm{PU}}_{i},\;Q\prime ,\;F,\;T3)$$

According to Equation (19), we apply Rule 6 to deduce the following:

(20) $$U_{i}\;|\equiv {\;S}_{j}\;|\equiv \;F$$

According to Hypothesis 16 and Equation (20), we apply Rule 3 to deduce the following:

(21) $$U_{i}\;|\equiv \;F$$

According to Hypothesis 12, 17–19 and Equation (21), we apply Rule 5 to deduce the following:

(22) $$U_{i}\;|\equiv {\;(\mathrm{PU}}_{i},\;Q,\;F,\;T3)$$

According to Equation (7) and Equation (16), we deduce the following:

(23) $$S_{j}|\equiv {\;((\mathrm{PU}}_{i}{,\;Q,\;F,\;T3)}_{h}{,\;\mathrm{PU}}_{i}{,\;\mathrm{SK}}_{U\;}\cdot {\;F)}_{h}{=\mathrm{SK}}_{\mathrm{ij}}$$

which satisfies Goal 3.

### 5.2. Formal Verification by Scyther Tool

The Scyther tool [30] is an automated, remarkable formal verification tool for inspecting protocol security against identity attacks. The standard version works based on the Dolev-Yao model [23]. As such, it probes protocol security by harnessing security assertions. Moreover, it corroborates all security assertion categories in the protocol, graphically engendering any incursions per assertion. The Scyther tool employs the security protocol description language (SPDL) for describing the model of the protocol under investigation.

Scyther also provides a graphical user interface. After the security claims, roles, and protocol have been specified, security validation begins by executing the authentication command. One important assumption of the model is that it espouses a black-box cryptographic model. With this tool, protocols are modeled based on role definition. Leveraging Scyther, both the correctness and the authenticity of security protocols can be examined. We therefore modeled the proposed protocol according to the SPDL to verify protocol claims.

**Figure 5:**
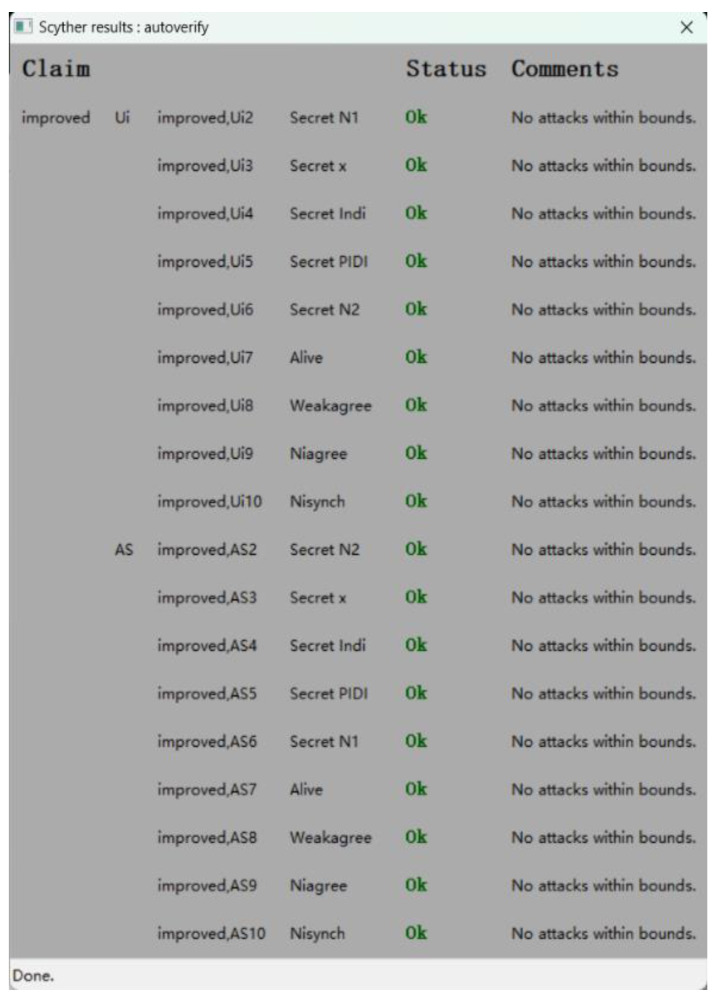
Verification of security claims between *U_i_* and *AS*.

**Figure 6:**
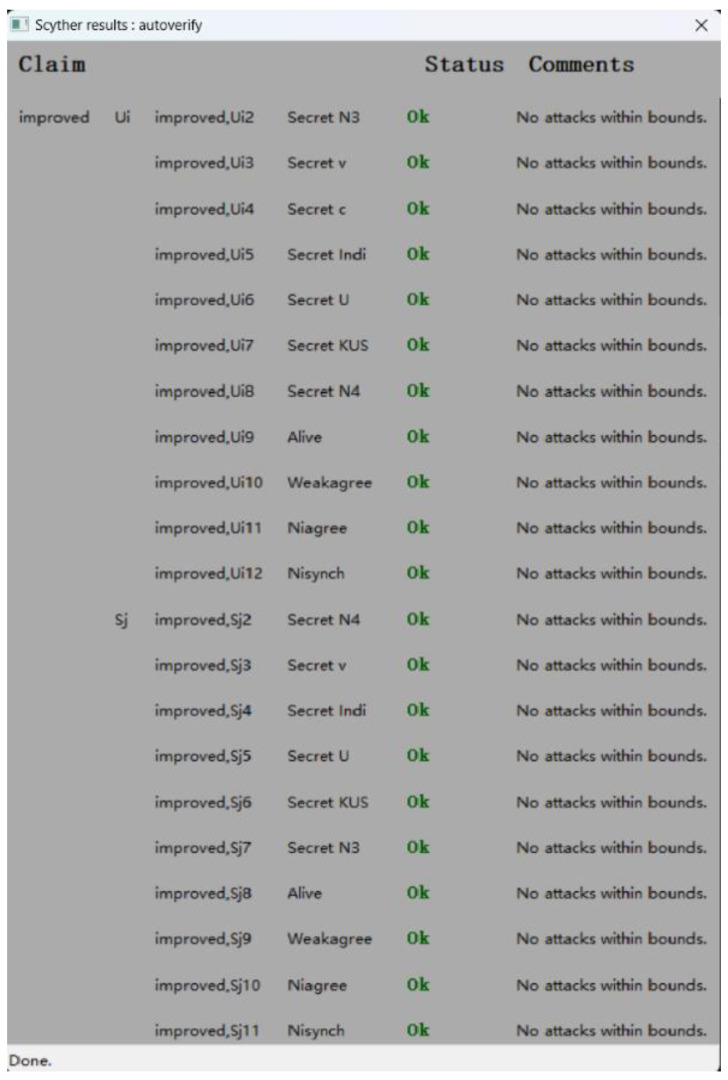
Verification of security claims between *U_i_* and *S_j_*.

The ASAP-IIOT is modeled according to the definition of the role of participants in the protocol and then these roles communicate to each other through *recv* and *send* channels. In this section, only the pre-authentication of user process and the mutual authentication process are verified because the process of the pre-authentication of server is similar to that of the pre-authentication of user. The verification results are shown in Figure 5 and Figure 6. The result for each claim is either valid with OK or invalid with Fail. For both user and server roles, all claims return OK without attacks found. For example, the validation of non-injective agreement (Niagree) ensures that the user and server agree on the content of exchanged messages, while the validation of non-injective synchronization (Nisynch) is a claim to ensure that the user and the server communicate following the same order of exchanged messages. The final result shows that the proposed ASAP-IIOT guarantees the secrecy of sensitive parameters like *N_1_, x,* and *Ind_i_*_._ In summary, these results show that the ASAP-IIOT scheme is secure to meet most of the security properties.

### 5.3. Security Analysis

This section provides an informal analysis of the security aspects of the proposed scheme. A summary of the security assessments for the ASAP-IIOT, in comparison to other relevant schemes, is presented in Table 3.

1. Replay attacks

In the ASAP-IIOT scheme, the *U_i_* and *S_j_* leverage random values including *N*_3_, *N*_4_, and timestamps to uphold message freshness among the participants in each session. It can circumvent clock asynchronization predicaments while fostering resilience against replay attacks.

2. Impersonation attacks

In order to impersonate the *S_j_*, the attacker *A* has to create a valid response as an authentication response {*Ah*, *F*, *T*3}, where *Ah* = *h*(*PU_i_*, *Q*′, *F*, *T*3). Since attacker *A* cannot compute *Ah* without having the secret values of *Q*′ = *SK_S_*⋯*R*, T3, and *U_i_*, which are generated through decryption of *E_U_* by the key of *K*′*_US_* = *SK_S_*⋯*PK_U_*, *A* has to generate a valid authentication request {*E_U_*, *R*, *T*1} to forge the identity of the user *U_i_*, where *R* = *N*_3_⋯*G*, *E_U_* = *E_Ku_*_s_(*c*, *PU_i_*, *Ind_i_*, *T*1), and *c* = *v* + *N*_3_ + *SK_U_*. However, *A* is not able to calculate valid corresponding *c* and *E_U_* because it lacks cognizance of *SK_U_*. Therefore, the *S_j_* can detect impersonation attacks by validating *c*. If *G = x*⋯*PK_AS_* + *R* + *PK_U_* + *Ind_i_*, it is verified. So, the ASAP-IIOT demonstrates comprehensive resilience against *U_i_* and *S_j_* impersonation assaults.

3. Modification attacks

In the authentication phase, if the *AS* sends {*Ah*, *F*, *T*3} to the *U_i_* and the attacker *A* changes the message {*Ah*, *F*, *T*3}, the *U_i_* can recognize this change by checking whether *Ah*′ = *Ah*. Since *Ah*′ is computed as *Ah*′ = *h*(*PU_i_*, *Q*, *F*, *T*3), where *PU_i_* = *SK_U_*⋯*Ind_i,_* and it is seen that the *PU_i_* can affect the value of *Ah*′, the ASAP-IIOT scheme has full capacity resistance to modification or manipulation attacks.

4. Man-in-the-middle attacks

Due to its capacity against modification or manipulation attacks, the ASAP-IIOT scheme has full ability to resist man-in-the-middle attacks. Since every party verifies the integrity of exchanged messages, any alterations will be discernible to the recipient.

5. Traceability attacks

In traceability attacks, messages with constant values may reveal the context or patterns of the communication. Thwarting such attacks necessitates message randomization via pseudorandom number integration. The proposed scheme incorporates ephemeral random secrets *N*_3_ and *N*_4_ to induce requisite message randomness on a per-session basis, thereby mitigating traceability incursions.

6. The property of non-traceability

Since attacker *A* cannot obtain any identifying information about the participants by listening to the messages delivered over the wireless channels in the pre-authentication phase, and the messages exchanged over the wireless channels in the authentication phase remain protected by the ECC and hash functions, the adversary is incapable of gleaning the information of the participants in the communication. Consequently, in the ASAP-IIOT scheme, the adversary tracing of the *U_i_* and *S_j_* could be prevented.

7. The property of user anonymity

In the proposed ASAP-IIOT scheme, an adversary cannot extract any static information pertaining to user *U_i_*′s identity, namely, *U_i_* and *Ind_i_*. Since the authentication request {*E_U_*, *R*, *T1*} comprises *Indi* and *U_i_* encrypted symmetrically by *K_US_*_,_ and the adversary lacks cognizance of *SK_U_*, *SK_S_*, and *K_US_* for decrypting *E_U_* and faces the ECCDHP for computing *K_US_*, user anonymity properties can be upheld by the ASAP-IIOT scheme.

8. The property of clock synchronization

According to the ASAP-IIOT, the *U_i_* and *S_j_* utilize random values and timestamps to ensure the freshness of exchanged messages within each session. By incorporating these random values and timestamps in the exchanged messages, the ASAP-IIOT effectively mitigates desynchronization issues, thereby circumventing problems associated with clock desynchronization.

9. The property of perfect secrecy

In the ASAP-IIOT, the session key generated by user *U_i_* during the authentication phase is represented as *SK_ij_* = *h*(*Ah*′, *PU_i_*, *SU*). Even if an attacker captures long-term keys like *K_US_*, they are unable to compute session keys used in previous sessions or those intended for future sessions. This is because the value of the session key depends on random values associated with the current session, such as *N*_3_ and *N*_4_. Attackers cannot retrieve *N_3_* and *N*_4_ separately from *R* and *F* through ECCDHP, ensuring both forward and backward secrecy features for the session key. Therefore, the ASAP-IIOT exhibits complete confidentiality, as concluded from the aforementioned context.

## 6. Performance Evaluation

**Figure 7:**
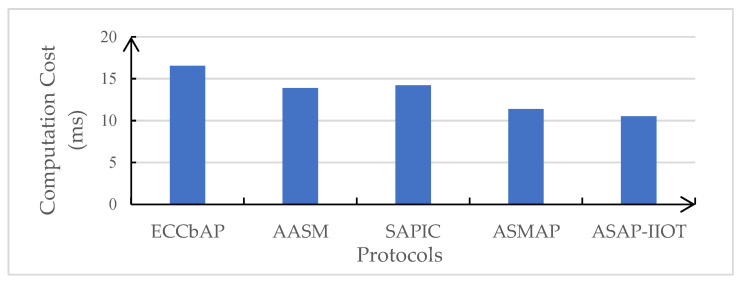
Comparison of computational costs.

**Figure 8:**
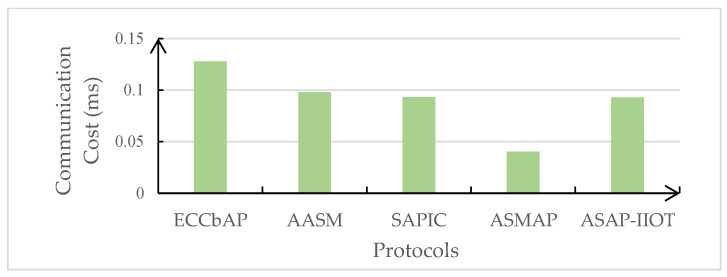
Comparison of communication costs.

**Figure 9:**
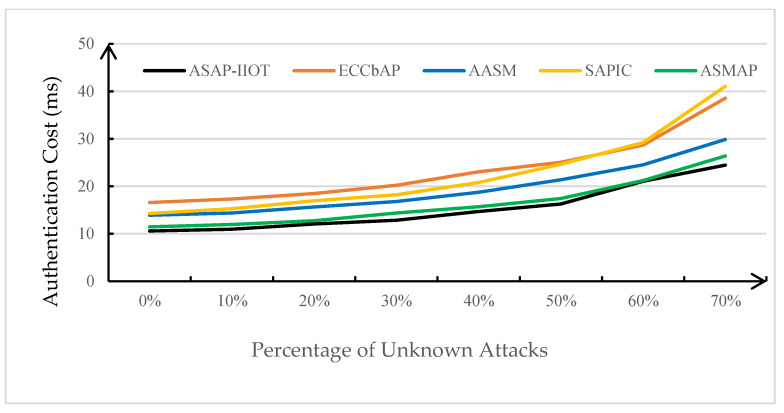
Average time taken for one successful protocol execution.

**Figure 10:**
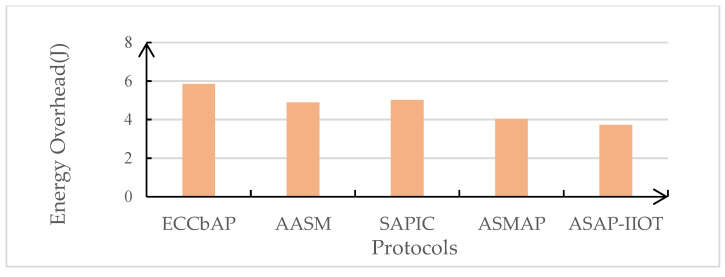
Energy consumed during a successful execution.

In this subsection, the performance of the ASAP-IIOT scheme is evaluated and compared with those of other state-of-the-art schemes including the ECCbAP scheme in [12], the AASM scheme in [14], the SAPIC scheme in [20], and the ASMAP scheme in [22] in terms of performance metrics such as computation costs, communication costs, total authentication overhead, and energy overhead. The performance evaluation is only conducted on the mutual authentication process to disclose the real-time features of the security schemes under the study. The results of these comparisons are shown in Table 4, Table 5, Figure 7, Figure 8, Figure 9, and Figure 10, respectively.

### 6.1. Computation Costs

We have established a testing environment to simulate the computational overhead of the cryptographic operations required in the ASAP-IIOT scheme and the other three schemes mentioned above.

The simulation experiments are conducted using the C/C++ OPENSSL library [31] and are performed on an Intel(R) Core (TM) i5-8300H with a CPU 2.30 GHz as the *U_i_*; it is assumed that the computational speed of the *S_j_* is twice of that of the *U_i_*. The execution times of the single bilinear function (*T_bp_*), the pairing hash function (*T_h_*), the symmetric encryption and decryption (*T_s_*), the scalar point multiplication based on ECC (*T_m_*), the multiplicative inverse operation (*T_i_*), the modular exponentiation operation (*T_e_*), and the ECC point addition (*T_a_*) are measured to be 5.275, 0.116, 0.002, 1.717, 0.001, 0.339, and 0.001 milliseconds (ms), while the XOR operation is trivial and can be ignored.

In the ECCbAP scheme, the user and server incur computational costs of (7*T_m_* + *T_a_*) and (6*T_m_* + *T_a_*), respectively. Consequently, the aggregate computational overhead for the ECCbAP scheme amounts to (13*T_m_* + 2*T_a_*). In the AASM scheme, the computational costs of the user and server are (4*T_m_* + 5*T_h_* + *T_a_* + *T_e_*) and (5*T_m_* + 5*T_h_* + 2*T_a_* + *T_bp_*), respectively. Therefore, its total computational cost equals (9*T_m_* + 10*T_h_* + 3*T_a_* + *T_e_* + *T_bp_*). In the SAPIC scheme, the computational costs of the user and server are (6*T_m_* + 2*T_a_* + *T_i_* + 2*T_h_*) and (5*T_m_* + *T_a_* + *T_i_* + 2*T_h_*), respectively. Therefore, its total computational cost equals (11*T_m_* + 3*T_a_* + 2*T_i_* + 4*T_h_*). In the ASMAP scheme, the computational costs of the user and server are *(*4*T_m_* + 4*T_h_*) and (4*T_m_* + 5*T_h_*), respectively. Consequently, the aggregate computational overhead for the ASMAP scheme amounts to *(*8*T_m_* + 9*T_h_)*. On the other hand, in the ASAP-IIOT scheme, the computational costs of (3*T_m_* + 2*T_h_* + 1*T_s_*) and (6*T_m_* + 3*T_h_* + 1*T_s_* + 3*T_a_*) are incurred by the user and server, respectively. The aggregate computational overhead for the ASAP-IIOT amounts to (9*T_m_* + 5*T_h_* + 2*T_s_* + 3*T_a_*), which is the lowest computational cost among the four schemes compared, at 10.539 ms.

### 6.2. Communication Costs

The communication costs should include transmission delay and propagation delay. It is assumed that the data transmission rate is 50 Mbps in 4G wireless LTE systems. It is assumed that 160 bits, 128 bits, and 32 bits are the requisite bit lengths of the hash function, SHA-1 [32], random numbers, and timestamps, respectively.

In the LTE system, the maximum distance from an eNodeB to a user is 1000 m. It is assumed that the maximum transmission distance in our device-to-device system is also 1000 m. The propagation speed is 3 × 10^8^ m/s. Since half of the maximum distance is reasonably assumed as the average distance, the average propagation cost is 1.7 µs. In the ECCbAP scheme, the SAPIC scheme, and the ASMAP, the user and the server will have three incidences of communication, so the total propagation cost is 5.1 µs. In the AASM scheme and the ASAP-IIOT scheme, the user and the server will have two incidences of communication, so the total propagation cost is 3.4 µs.

In the ECCbAP scheme, since the user transmits an authentication message {*PPID_i_*, *P*1, *P*2, *V_i_*} to the server with total information of 4096 bits, the transmission delay is 0.08192 ms, causing the communication cost of the user to be 0.08532 ms. Additionally, since the server response comprises {*P*3, *P*4} encapsulating 2048 bits of information, the transmission delay of the server is 0.04096 ms, causing the communication cost of the server to be 0.04266 ms. Therefore, the ECCbAP scheme incurs a total communication cost of 0.12798 ms. In the AASM scheme, the user transmits an authentication message {*M*, *N*, *σ*, *T_u_*} to the server with 2496 bits of information. The transmission delay of the user is 0.04992 ms, causing the communication cost of the user to be 0.05162 ms. Then, the server transmits a message {*t*, *Y*, *T_ms_*} to the user of 2240 bits in length. The transmission delay of the server is 0.0448 ms, causing the communication cost of the server to be 0.0465 ms. Consequently, the total transmission delay is 0.09472 ms, while the total communication cost is 0.09812 ms. In the SAPIC scheme, the user transmits an authentication message {*P*1, *P*2, *E_SK_*[*H*(S*K*)]} to the server, while the server transmits an authentication message {P*3*, *P*4} to the user. Each of *P*1, *P*2, *P*3, and *P*4 is 1024 bits in length, and *E_SK_*[*H*(*SK*)] requests 320 bits in length. The transmission delay of the user is 0.04736 ms, while the communication cost of the user is 0.05076 ms. The transmission delay of the server is 0.04096 ms, causing the communication cost of the server to be 0.04426 ms. The total transmission delay is 4 *ECC* + *ESK*[*H*(*SK*)] = 0.08832 ms, while the total communication cost is 0.09342 ms. In the ASMAP scheme, the user transmits an authentication message {*P*_1_, *P*_2_, *EI_i_*, *V_i_*} to the server with 960 bits of information. The transmission delay of the user is 0.0192 ms, causing the communication cost of the user to be 0.0226 ms. Then, the server transmits a message {*P*_3_, *P*_4_, *T*′*_i_*} to the user of 800 bits in length. The transmission delay of the server is 0.016 ms, causing the communication cost of the server to be 0.0177 ms. Consequently, the total transmission delay is 0.0352 ms, while the total communication cost is 0.0403 ms.

In the ASAP-IIOT scheme, the user transmits {*EU*, *R*, *T*1} to the server, where *R* constitutes elliptic curve group elements encapsulating 1024 bits of information, *T*1 occupies 32 bits, *EU* = *EKus*(*c*, *Ui*, *Indi*, *T*1) with *Ui* = *N*2⋯*PKU*, and *Indi* is elliptic curve group points of 1024 bits each, while *c* ∈ Zq* occupies 128 bits. Consequently, this request holds 3264 bits of information. Therefore, the transmission delay of the user is 0.06528 ms, causing the communication cost of the user to be 0.06698 ms. Additionally, the server returns an authentication response {*Ah*, *F*, *T*3} to the user, where *Ah* = *h*(*Ui*, *Q*′, *F*, *T*3) requests 160 bits, *F* spans 1024 bits, and *T*3 occupies 32 bits, adding up to (160 + 1024 + 32 = 1216) bits. Therefore, the transmission delay of the server is 0.02432 ms, causing the communication cost of the server to be 0.02602 ms. The total transmission delay is 0.0896 ms, while the total communication cost is 0.093 ms.

### 6.3. Authentication Overheads

Authentication overhead is normally the sum of the computation cost and communication cost. However, any protocol may face various types of attacks, broadly categorized as known and unknown attacks. The known attacks are the attacks which have been ascertained to be thwarted by a security scheme and their occurrence cannot break the operation of the protocol without an impact on the protocol’s execution time. On the other hand, unknown attacks have the potential to disrupt the protocol’s operation and produce negative impacts on the execution time of a security protocol because they could be new malicious attacks or they are not necessarily prevented by a security protocol. With the consideration of coexistence of the known and unknown attacks, a performance evaluation on the robustness of the four protocols in comparison has been conducted following the method reported in [33,34,35]. Analyzing the impact of these unknow attacks, it is assumed that the probability of an unknown attack occurring at the *i*-th step is $q=\frac{1}{n}$, where *n* represents the total number of operational steps in a protocol. Therefore, the average time required for a successful execution of the protocol can be calculated as follows:

$$t_{\mathrm{avg}}=\frac{\Sigma_{i=1}^{n}{\;q\;\times \;t}_{\mathrm{fail}\_i}{\;\times \;p+t}_{\mathrm{suc}\;}\times \;(1-p)}{1-p}\;$$

where *p* represents the proportion of unknown attacks to total attacks, *t_suc_* represents the time cost when no attack occurs, and *t_fail_i_* indicates the total time cost before an attack occurs in the *i*-th step. As shown in Figure 9, as the proportion of unknown attacks gradually increases, there is a rising trend in the average time required for successful execution of the protocol. Compared to the ECCbAP scheme, the AASM scheme, the ASMAP scheme, and the SAPIC scheme, the ASAP-IIOT scheme takes the lowest average time to successfully complete an authentication process under known and unknown attacks. Moreover, as the proportion of unknown attacks increases, the ASAP-IIOT solution exhibits a relatively small variation in the average time required for a successful execution.

### 6.4. Energy Overhead

The energy is utilized based on the formula *E* = *V* ∗ *I* ∗ *T*, where voltage (*V*), current drawn (*I*), and the operation time (*T*) are the contributing factors. In our system, *V* = 220 *V* and *I* = 1.6 A. Figure 10 illustrates the energy consumed during a single successful execution of the protocol between the proposed scheme and the corresponding existing scheme. The highest energy overhead is that of the ECCbAP scheme at 5.84 J. The energy consumption of the SAPIC scheme is 5.03 J, the AASM scheme consumes 4.9 J, and the ASMAP scheme consumes 4.04 J. The proposed scheme incurs the lowest energy overhead of 3.73 J for the IoT device.

## 7. Further Discussions

The proposed protocol has advantages in both security functionality and system performance. It is a lightweight solution compared with other benchmark protocols. The ASAP-IIOT exhibits the best performance in the scenarios with or without network attackers compared to other protocols. The study results demonstrate that ASAP-IIOT’s overhead is more favorable than alternative protocols.

However, implementing the ASAP-IIOT scheme may present another challenge. While we have tested the robust performance of our protocol under simulated attacks, considering the infrastructure of real-world networks that connect a large number of devices, whether frequent user data storage poses certain security risks requires extensive testing and simulation. Additionally, the security of the proposed protocol depends on the robustness of the cryptographic key exchange function ECDLP. The emergence of quantum computing attacks poses a threat to the security of traditional encryption algorithms, including ECDLP. Therefore, future research needs to focus on developing post-quantum cryptographic techniques to resist quantum computing attacks.

Furthermore, the proposed ASAP-IIOT scheme works at the data link layer to protect the operations of the IIoT systems from various malicious protocol attacks. However, in the implementation of this scheme, there could be some physical layer attacks to impair the communication among the devices in the system. Those possible physical layer attacks may include side-channel attacks (SCA) on the ECC, simple power analysis (SPA) on the ECC, and differential power analysis (DPA) on the ECC, aiming to exploit various leaked information generated during hardware execution to obtain ciphertext information. We are fully aware of the severe threats generated by those physical layer attacks on the operation of the IIoT systems. Since the proposed ASAP-IIOT solution cannot resist the physical layer attacks, some existing solutions can be found in [36,37] to eliminate them.

## 8. Conclusions

In this paper, a lightweight ECC-based authentication and key agreement protocol ASAP-IIOT has been developed for mutual authentication between users and application servers. The logical correctness of the protocol has been proven by BAN logic, while the security functionality of the ASAP-IIOT scheme has been validated by using the Scyther tool, corroborated by qualitative security analysis. It has been demonstrated that compared to other relevant schemes, the ASAP-IIOT scheme can overcome most security vulnerabilities with reasonable computational, communication, and authentication costs. The overhead analysis indicates that among the referenced schemes compared, the ASMAP scheme shows the lowest overhead, while in comparison to the ASMAP scheme, our proposed solution demonstrates reductions of 0.31 J in energy and 0.8773 ms in authentication overheads. It shows that the ASAP-IIOT scheme is more suitable and feasible to be deployed in future IIoT systems.

## Figures and Tables

**Table 1:** Summary of relevant authentication protocols.

Literature	Authentication Scheme	Advantages	Disadvantages
Rostampour et al. [12]	ECCbAP	Provides perfect forward secrecy. Resistance to impersonation attacks, modification attacks, and replay attacks.	Computational overhead is a little high. Clock desynchronization problem. Affected by traceability attacks.
Jia et al. [14]	AASM	Avoids clock desynchronization problem. Resistance to impersonation attacks, man-in-the-middle attacks, and replay attacks. Provides perfect forward secrecy.	Affected by modification attacks. Affected by traceability attacks.
Iqbal et al. [20]	SAPIC	Resistance to replay attacks, impersonation attacks, traceability attacks, modification attacks, and man-in-the-middle attacks.	The absence of the property of perfect secrecy. Clock desynchronization problem. Computational overhead is a little high.
Panda et al. [22]	ASMAP	Avoids clock desynchronization problem. Resistance to impersonation attacks, replay attacks, password-guessing attacks, and man-in-the-middle attacks. Provides perfect forward secrecy.	Affected by modification attacks.

**Table 2:** The notations used.

Notion	Description
*AS*	Authentication server
*S_j_*	The jth application server
*U_i_*	The ith use
*p*	Prime number in the finite field
*E*	Elliptic curve with order of p
*G_E_*	An additive group with order of q
*G*	Generator of *G_E_*
(·)	Scalar point multiplication based on ECC
*h*()	Hash function
Z_q_*	Integers {1, 2, 3, …, q−1} in the finite field
*ID_i_*	Identity of user *U_i_*
*SK_ij_*	The session key between *U_i_* and *S_j_*
Δ*T*	Maximum communication transmission delay
*T*1, *T*2, *T*3, *T*4	The current timestamps
*K_US_*	The shared secret key between the *U_i_* and *S_j_*
(*SK_AS_*, *PK_AS_*)	Private/public key pair of authentication server
(*SK_S_*, *PK_S_*)	Private/public key pair of application server
(*SK_U_*, *PK_U_*)	Private/public key pair of user
*A*	Adversary

**Table 3:** Security feature comparison of the ASAP-IIOT with other protocols.

Security Feature	ECCbAP	AASM	SAPIC	ASMAP	ASAP-IIOT
Resistance to replay attacks	✓	✓	✓	✓	✓
Resistance to impersonation attacks	✓	✓	✓	✓	✓
Resistance to modification attacks	✓	×	✓	×	✓
Resistance to modification attacks	✓	✓	✓	✓	✓
Resistance to traceability attacks	×	×	✓	✓	✓
The property of non-traceability	×	×	✓	✓	✓
The property of user anonymity	×	✓	✓	✓	✓
The property of clock synchronization	×	✓	×	✓	✓
The property of perfect secrecy	✓	✓	×	✓	✓

**Table 4:** Comparison of computational costs.

	User (ms)	Server (ms)	Total Cost (ms)
ECCbAP	11.559	4.9705	16.5295
AASM	7.118	6.768	13.886
SAPIC	9.995	4.2075	14.2025
ASMAP	7.342	4.064	11.406
ASAP-IIOT	5.311	5.228	10.539

**Table 5:** Comparison of communication costs.

	User (ms)	Server (ms)	Total Cost (ms)
ECCbAP	0.08532	0.04266	0.12798
AASM	0.05162	0.0465	0.09812
SAPIC	0.05076	0.04266	0.09342
ASMAP	0.0226	0.0177	0.0403
ASAP-IIOT	0.06698	0.02602	0.093

## Data Availability

No new data were created or analyzed in this study. Data sharing is not applicable to this article.
